# Sequentially calibrating a Bayesian microsimulation model to incorporate new information and assumptions

**DOI:** 10.1186/s12911-021-01726-0

**Published:** 2022-01-12

**Authors:** Maria DeYoreo, Carolyn M. Rutter, Jonathan Ozik, Nicholson Collier

**Affiliations:** 1 RAND Corporation, 1776 Main St., Santa Monica, CA 90401 USA; 2 Argonne National Laboratory, Building 221, 9700 South Cass Avenue, Argonne, IL 60439 USA

## Abstract

**Background:**

Microsimulation models are mathematical models that simulate event histories for individual members of a population. They are useful for policy decisions because they simulate a large number of individuals from an idealized population, with features that change over time, and the resulting event histories can be summarized to describe key population-level outcomes. Model calibration is the process of incorporating evidence into the model. Calibrated models can be used to make predictions about population trends in disease outcomes and effectiveness of interventions, but calibration can be challenging and computationally expensive.

**Methods:**

This paper develops a technique for sequentially updating models to take full advantage of earlier calibration results, to ultimately speed up the calibration process. A Bayesian approach to calibration is used because it combines different sources of evidence and enables uncertainty quantification which is appealing for decision-making. We develop this method in order to re-calibrate a microsimulation model for the natural history of colorectal cancer to include new targets that better inform the time from initiation of preclinical cancer to presentation with clinical cancer (sojourn time), because model exploration and validation revealed that more information was needed on sojourn time, and that the predicted percentage of patients with cancers detected via colonoscopy screening was too low.

**Results:**

The sequential approach to calibration was more efficient than recalibrating the model from scratch. Incorporating new information on the percentage of patients with cancers detected upon screening changed the estimated sojourn time parameters significantly, increasing the estimated mean sojourn time for cancers in the colon and rectum, providing results with more validity.

**Conclusions:**

A sequential approach to recalibration can be used to efficiently recalibrate a microsimulation model when new information becomes available that requires the original targets to be supplemented with additional targets.

## Background

Microsimulation models (MSMs) are mathematical models that simulate event histories for individual members of a population. They can be used to simulate a large number of individuals from an idealized population, with features that change over time. MSMs are useful for policy decisions because life histories can be simulated under alternative scenarios or assumptions, and aggregated to predict population-level outcomes. MSMs can be used to estimate effects of interventions that may influence multiple components of the model in complex ways [9].

Health policy MSMs represent social or biological mechanisms that determine health or economic outcomes and rely on assumptions about how policy choices will affect those outcomes [25]. Measures or outcomes that are relevant to the health policy question at hand can then be summarized by aggregating individual-level predictions to understand the effects of different policies. MSMs have been applied to health policy questions since 1985 [44]. Cancer is one area of application where MSMs have been heavily used to inform policy, with the U.S. Preventive Services Task Force considering the effectiveness of different screening regimens predicted by MSMs when developing guidelines for breast [22], cervical [14], colorectal [17], and lung cancer [4].

Information may be directly available on some parameters that govern the model predictions, such as the sensitivity and specificity of a screening exam or survival time following diagnosis. Such parameters are referred to as inputs, which are fixed rather than estimated. However, most parameters will be related to observable outcomes or available evidence in a complex way. For example, population-level outcomes such as colorectal cancer (CRC) incidence rates can be observed from national cancer registries, but these are functions of the unobservable process of developing CRC, which depends on the rate of adenoma (precursor lesion) initiation, and the growth and transition to preclinical (asymptomatic) and clinical (detected) cancer. The functional relationships between the observed data and the MSM parameters is not known. Evidence provided by screening trials and epidemiological studies informs MSM parameters, but the function of this relationship is not known and parameters may interact in complex ways to generate the observed outcomes.

At its simplest, model calibration is the process of incorporating evidence into the model [25], and is accomplished by selecting parameter values that result in model predictions that are consistent with observed or expected results based on observed data or expert knowledge. Calibrated MSMs can be used to make predictions about population trends in disease outcomes and effectiveness of interventions. However, calibration can be a challenging and time consuming process because it involves searching a high dimensional parameter space to predict many targets simultaneously, and it is sometimes difficult to find suitable parameter values. There are many methods for calibration, ranging from simple one-at-a-time parameter perturbation [37], to grid search algorithms from engineering [15, 19], which may break down when calibrating a large number of parameters. Directed searches use the derivative of the likelihood function, or an approximation to the derivative, to move toward areas of improved fit (e.g., [30, 35]), however for MSMs, the likelihood function is generally not available in closed form.

Bayesian approaches to calibration are particularly useful in the context of MSMs for policy-making because they combine different sources of evidence and enable estimation and uncertainty quantification of parameters, functions of parameters, and model predictions. Such uncertainty arises from variability in calibration data (sampling variability), simulation (Monte Carlo) variability, and parameter estimation. Markov Chain Monte Carlo (MCMC) is a commonly used class of algorithms for estimating the parameters of a Bayesian model [41, 50]. Rutter et al. [41] developed an approximate Metropolis-Hastings algorithm that includes an embedded simulation to estimate the likelihood function used to calculate the acceptance ratio. The MCMC algorithm was used to simulate draws from the posterior distribution of the parameters of a MSM for CRC given calibration targets. However, MCMC can be costly to apply to MSM calibration because the likelihood is often intractable and computationally expensive to estimate [25], and because it is based on a process of sequentially updating draws. The calibration process can also require a large number of samples from a model’s parameter space to be used for propagating uncertainties of the resulting parameter estimates, which can make certain non-parametric, Gaussian process-based Bayesian approaches less feasible [8].

In order to take advantage of modern computing resources, Rutter et al. [43] developed a likelihood-free approximate Bayesian computation (ABC) algorithm [23, 46], referred to as Incremental Mixture ABC (IMABC), that was successfully applied to calibrate a MSM for CRC, referred to as CRC-SPIN 2.0 (ColoRectal Cancer Simulated Population model for Incidence and Natural history). Once CRC-SPIN 2.0 had been calibrated, posterior distributions were examined, and predictions from the model were compared to external validation targets that were not used in model calibration [40]. This examination revealed that very little information on sojourn time, the time from initiation of preclinical cancer to presentation with clinical CRC, was provided by the calibration targets. The calibrated model also resulted in screen detected cancer rates (the percentage of patients with cancer detected via colonoscopy screening) that were too low and indicated that additional calibration targets should be incorporated to better inform this aspect of the disease process. One approach is to perform another full calibration, including one or more new targets in addition to the previous set. However, this is time consuming and does not build on information about parameters obtained from the original calibration. This paper develops a technique for sequentially updating MSMs to take full advantage of earlier calibration results that are based on a similar set of targets, with the objective being to ultimately speed up the calibration process.

The remainder of this paper is organized as follows. In “Calibrating the microsimulation model” section, we present the CRC-SPIN 2.0 model, the calibration targets included, and the resulting estimates of parameters and predictions arising from this calibrated model. In “Recalibrating the microsimulation model to incorporate new study targets” section, we explore methods for incorporating additional (new) calibration targets and updating the parameter draws so that the model calibrates to the new set of calibration targets. “Discussion” section concludes with a discussion of other applications of this method, implications for modelers and remaining questions to be addressed in future work.

## Calibrating the microsimulation model

### The microsimulation model for colorectal cancer

The ColoRectal Cancer Simulated Population Incidence and Natural history model [41, 42] describes the natural history of CRC based on the adenoma-carcinoma sequence [20, 26]. Four model components describe the natural history of CRC: (1) adenoma risk; (2) adenoma growth; (3) transition from adenoma to preclinical cancer; and (4) transition from preclinical to clinical cancer (sojourn time). CRC-SPIN has been used to provide guidance to the Centers for Medicare and Medicaid Services (CMS) [51] and to inform U.S. Preventive Services Task Force CRC screening guidelines [17]. We provide an overview of CRC-SPIN 2.0 [43], which contains 22 calibrated parameters, $\theta$, and prior distributions which are truncated normal or uniform, as informed by prior knowledge from published literature and previous calibration exercises. We refer the reader to [43] and online at cisnet.cancer.gov [28] for more detail.

#### Adenoma risk

Adenomas are assumed to arise according to a non-homogeneous Poisson process with a piecewise linear age-effect. The *i*th agent’s baseline instantaneous risk of an adenoma at age $a=20$ years is given by $\psi _i(20)= \exp (\alpha _{0i} + \alpha _1 \text{ female}_i$) where $\alpha _{0i} \sim N(A,\sigma _\alpha )$ and $\alpha _1$ captures the difference in risk for women (female$_i=1$ indicates agent *i* is female). Adenoma risk changes over time, generally increasing with age, a process we model using a piecewise linear function for log-risk with knots at ages 50, 60, and 70 and assuming zero risk before age 20 [18]:

1 $$\begin{aligned} \ln (\psi _i(a))= & {} \alpha _{0i} + \alpha _1 \text{ female}_i + \delta (a\ge 20)\min (a-20,30)\alpha _{20} \nonumber \\&+\delta (a\ge 50)\min ((a-50),10)\alpha _{50} \nonumber \\&+ \delta (a\ge 60)\min ((a-60),10)\alpha _{60} \nonumber \\&+ \delta (a\ge 70)(a-70)\alpha _{70} . \end{aligned}$$

#### Adenoma growth

For each adenoma, we simulate a hypothetical time to reach 10mm, assuming that $t_{10mm}$ has a Frèchet distribution with shape parameter $\beta _1$, scale parameter $\beta _2$, and cumulative distribution function given by

2 $$\begin{aligned} F(t) = \exp \left[ - \left( \frac{t}{\beta _2} \right) ^{-\beta _1} \right] \end{aligned}$$

for $t \ge 0$. We allow different scale and shape parameters for adenomas in the colon and rectum, using the notation $\beta _{1c}$ and $\beta _{2c}$ for the colon, and $\beta _{1r}$ and $\beta _{2r}$ for the rectum.

Adenoma size at any point in time is simulated using the Richard’s growth model, with a calibrated parameter that allows for a wide range of sigmoidal growth patterns [48]. The diameter of the *j*th adenoma in the *i*th agent at time *t* after initiation is given by

3 $$\begin{aligned} d_{ij}(t) = d_\infty \left[ 1 + \left( \left( \frac{d_0}{d_\infty }\right) ^{1/p} -1 \right) \exp (-\lambda _{ij} t) \right] ^p \end{aligned}$$

where $d_0=1$ mm is the minimum adenoma diameter in millimeters (mm) and $d_\infty =50$ is the maximum adenoma diameter. The calibrated parameter *p* determines the shape of the growth curve. The growth rate for the *j*th adenoma within the *i*th agent, $\lambda _{ij}$, is calculated by setting $t=t_{\mathrm{10\,mm}}$ and $d_{ij}=10$ in Eq. (3).

#### Transition from adenoma to preclinical invasive cancer

For the *j*th adenoma in the *i*th agent, the size at transition to preclinical cancer (in mm) is simulated using a log-normal distribution; the underlying (exponentiated) normal distribution is assumed to have standard deviation $\sigma _{\gamma }$ and mean

4 $$\begin{aligned} \mu _{ij} = \gamma _0 + \gamma _1 \text{ female}_{i} + \gamma _2 \text{ rectum}_{ij} + \gamma _3 \text{ female}_{i} \text{ rectum}_{ij} + \gamma _4 \text{ age}_{ij} + \gamma _5 \text{ age}_{ij}^2 \end{aligned}$$

where rectum$_{ij}$ is an indicator of rectal versus colon location and age$_{ij}$ is the age at adenoma initiation in decades, centered at 50 years. Based on this model, the probability that an adenoma transitions to preclinical cancer increases with size. Most adenomas do not reach transition size and small adenomas are unlikely to transition to cancer.

#### Sojourn time

Sojourn time is the time between the transition from preclinical (asymptomatic) CRC to clinical (symptomatic and detected) cancer. We simulate sojourn time using a Weibull distribution with survival function

5 $$\begin{aligned} S(t) = \exp \left( -\left( \frac{t}{\lambda _1}\right) ^{\lambda _{2}} \right) \end{aligned}$$

for preclinical cancer in the colon, and assume a proportional hazards model, with hazard ratio $\exp (\lambda _3\text{ rectum}_{ij})$, to allow sojourn time to systematically differ for preclinical cancers in the colon and rectum.

#### Simulation of lifespan and colorectal cancer survival

Once a cancer becomes clinically detectable, we simulate stage and tumor size at clinical detection based on Surveillance, epidemiology, and end results (SEER) data from 1975 to 1979, the most recent period prior to widespread dissemination of CRC screening tests [27]. Survival time after CRC diagnosis is based on the first diagnosed CRC and depends on age, sex, cancer location, and stage, and is simulated using relative survival estimates from analysis of SEER data from individuals diagnosed with CRC from 1975 through 2003 [39]. We assume proportional hazards of CRC and other-cause mortality within sex and birth-year cohorts. Other-cause mortality is modeled using survival probabilities based on product-limit estimates for age and birth-year cohorts from the National Center for Health Statistics Databases [29].

### Calibration data

Calibration data consist of individual-level data that are reported in aggregate in published studies. Calibration targets therefore take the form of summary statistics. Because targets come from small and larger studies, as well as registry data that results in very precisely estimated targets, the level of uncertainty varies across targets. Generating calibration targets requires simulating a set of agents with risk that is similar to the study population based on age, gender, prior screening patterns, and the time period of the study, which may affect both overall and cancer-specific mortality. This simulation can be computationally demanding, depending on the number of agents and the process used to simulate the particular target. We calibrated to 40 targets from six sources: SEER registry data ([27], 20 targets) and five published studies (20 targets). Let $y=(y_1,\dots ,y_{J})$ denote these $J=40$ calibration targets.

SEER colon and rectal cancer incidence rates in 1975–1979 are reported per 100,000 individuals and are a key calibration target. We assumed that the number of incident CRC cases in any year follows a binomial distribution with number of trials equal to the SEER population size. To simulate SEER incidence rates, we generated a population of individuals from aged 20 to 100 who are free from clinically detected CRC, with an age- and sex-distribution that matches the SEER 1978 population. Model-predicted CRC incidence is based on the number of people who develop CRC in the next year.

To simulate additional targets from published studies, we generated separate populations for each study that match the age and gender distribution of the sample during the time-period of the study. We assume that study participants are free from symptomatic (clinically detectable) CRC and have not been screened for CRC prior to the study. This is a reasonable assumption because studies used for model calibration were conducted prior to widespread screening, or were based on minimally screened samples.

Simulation of targets also requires simulating the detection and removal of adenomas and preclinical cancers. The probability of detection, or test sensitivity, is a function of lesion size, and is informed by back-to-back colonoscopy studies [10, 38]. We specify the probability of not detecting (or missing) an adenoma of size *s* that produces miss rates that are consistent with observed findings [10, 38] and were successfully used in [43].

The miss rate functions result in sensitivities of 0.81 for adenomas of 5mm, 0.92 for adenomas of 10mm, and 0.98 for adenomas of 15mm. For preclinical cancers, we assume sensitivity that is the maximum of 0.95 and sensitivity based on adenoma size, so that colonoscopy sensitivity is 0.95 for preclinical cancers 12mm or smaller, and sensitivity is greater than 0.95 for larger preclinical cancers.

### Calibration results and motivation for recalibration

A Bayesian approach to model calibration is preferred in this context to enable uncertainty quantification in model parameters and because of the ability to incorporate different sources of information through calibration targets and prior distributions. We use the Incremental Mixture Approximate Bayesian Computation (IMABC) algorithm developed by [43] and used successfully to calibrate the CRC-SPIN model with 22 parameters and 40 targets. We refer the reader to the Additional file 1 for more description of the algorithm. This algorithm generates a sample of MSM parameter draws that are from an approximate posterior distribution. This approach is an approximate Bayesian version of adaptive importance sampling [36, 47] and similar to the Population Monte Carlo ABC algorithm of Beaumont et al. [1]. While While McKinley et al. [24] showed that popular ABC methods can be inefficient or fail to converge when applied to complex, high-dimensional models (in their example there are 22 parameters and 18 outputs, which we refer to here as targets), the Incremental Mixture Approximate Bayesian Computation (IMABC) algorithm successfully calibrated the CRC-SPIN model with 22 parameters and 40 targets.

The application of IMABC in the experiments described here was implemented using the EMEWS framework [31] and run on the Midway2 cluster at the University of Chicago Research Computing Center and the Bebop cluster at Argonne National Laboratory. To take advantage of parallel processing, we used 80 node job allocations to execute up to 318 concurrent CRC-SPIN instances.

Internal model validation indicated that, as expected, the model simulated (predicted) targets were within the tolerance intervals of the observed calibration targets. However, the posterior distributions for two parameters of the sojourn time (time spent in the preclinical cancer phase) distribution largely reflected the prior distributions [43], and suggested too little time spent in the preclinical cancer phase. This finding was also noted by Rutter et al. [40] for an earlier version of the CRC-SPIN model, based on the results of a model validation, and comparison to other CRC models [16, 49].

We hypothesize that sojourn time is not well estimated because sojourn time is informed by screening studies, and our targets include only a single calibration target from a screening study that is imprecise and therefore has extremely wide tolerance intervals [11]. Sojourn time and preclinical cancer detection rates are closely related, as a longer sojourn time implies more time to detect preclinical cancers. In light of this finding, we sought to include an additional calibration target that would provide more information about sojourn time.

## Recalibrating the microsimulation model to incorporate new study targets

The United Kingdom Flexible Sigmoidoscopy Screening (UKFSS) Trial [12] was a large, randomized controlled trial that examined the effectiveness of a one-time flexible sigmoidoscopy. All adults aged between 55 and 64 years who were registered with participating practices were eligible to take part unless they met standard exclusion criteria. Eligible, interested respondents were randomly allocated to the screening or control groups of the trial. No screening program was in place in the UK during the period under study, so it is reasonable for the model to make the assumption of no prior screening for trial participants. One key baseline outcome reported from this study is percentage of patients with cancers detected upon screening. The rate of CRC detection was 0.0046 for men (derived from a sample size of 20,519 men) and 0.0017 for women (derived from a sample size of 20,155 women). This corresponds to an overall screen detection rate of 0.0032 based on 40,674 people. The previously included screen detection rate (the Imperiale target) was based on a small study that included only 1994 people, hence the UKFSS study is based on a sample size of more than 20 times the size, and therefore provides a much more precisely estimated target that will yield more information for estimating the model parameters.

### Methods

To revise the set of calibration targets and include a more precisely estimated set of targets (this is equivalent to replacing the imprecise target with the new targets since in this case the tolerance or confidence intervals around the precisely estimated target are fully contained in the other set), one option is to “start from scratch”, applying the IMABC algorithm or a different algorithm to calibrate the model to this revised set of targets. However, this may be inefficient and does not take advantage of what has already been learned about model parameters from the first calibration. An alternative is to build from the original calibration results, and update them in light of new information.

Let $z=(z_1,\dots ,z_{K})$ denote the new *K* calibration targets, to be added to the original targets *y*. We want to simulate from the posterior distribution of $\theta$, given original targets *y* and new targets *z*. Note that $p(\theta \mid y,z)$ is proportional to $p(\theta )p(y\mid \theta )p(z\mid \theta ,y)$, or $p(\theta \mid y)p(z\mid \theta ,y)$, where $p(\theta \mid y)$ is the posterior distribution from the last calibration, by standard Bayesian updating rules.

We explore two different approaches to calibration, the first being a “start from scratch” method that incorporates all of the original targets as well as the new gender-specific screen detection rate targets, and the second being a sequential calibration that begins with the current set of posterior samples and updates them to fit the new targets (as well as the original ones). The structure of the IMABC algorithm makes it amenable to a “warm start”. Because we have already successfully applied the IMABC algorithm to obtain samples from an approximate posterior fit to *y*, our sequential approach continues to iterate through the IMABC algorithm until the target ESS of 5000 is reached. For comparative purposes, the start from scratch method begins the IMABC algorithm in the standard way by drawing from the prior. The idea behind this sequential algorithm is that, although none of the posterior samples from the original calibrated model fit the gender-specific screen-detection targets well (e.g., none of the current samples are from the full posterior $p(\theta \mid y,z)$), they are still informative about where the posterior lies, since they are samples from $p(\theta \mid y)$.

Let the posterior distribution approximated by the original calibration be denoted by $p_{orig}(\theta \mid y)$. The start from scratch method and sequential calibration method are both used to calibrate the model to targets *y* and *z*, where *z* represents the more precise screen-detection rates. Let these posteriors be denoted by $p_{scratch}(\theta \mid y,z)$ and $p_{seq}(\theta \mid y,z)$. We focus on two main comparisons. The first is to compare $p_{orig}(\theta \mid y)$ to $p_{scratch}(\theta \mid y,z)$ and then determine whether, and to what degree, adding in new study targets *z* changes the model parameter estimates. The second is to compare $p_{scratch}(\theta \mid y,z)$ and $p_{seq}(\theta \mid y,z)$ to assess whether the method applied for recalibration, e.g., the start from scratch and sequential approaches, yield the same estimated posterior distributions.

To facilitate these comparisons, we compute a 95% credible interval overlap measure (e.g., [5, 13]) for each parameter and each pair of posterior distributions (Table 2). The overlap measure based on two (overlapping) credible intervals $(L_1,U_1)$ and $(L_2,U_2)$ is computed as $0.5(U_I-L_I)/(U_1-L_1)+0.5(U_I-L_I)/(U_2-L_2)$, where $(L_I,U_I)$ is the intersection of the two overlapping intervals. When the intervals are identical, this is equal to 1. When the intervals do not overlap at all, the measure is defined as equal to 0. This overlap measure averages the proportion of the first interval that is contained in the second, and the proportion of the second interval that is contained in the first. We also estimate the overlapping area between the two empirical posterior distributions (e.g., [6, 32, 41]). Values closer to 1 indicate more similar empirical distributions and values closer to 0 indicate the distributions overlap very little. We compute the standardized difference in means (SMD), which we define as the difference in posterior means, standardized by the estimated posterior standard deviation from the original calibration. Finally, we calculate an estimate of the Hellinger distance between the two distributions, using the “statip” package in R [34].

### Results

The start from scratch approach required 73 iterations and 737,715 simulated parameters to reach the target ESS of 5000. Starting from the previous iterations, the sequential approach required 50 iterations and 500,500 simulated parameters to reach the target ESS, which translates to a significant time savings given that microsimulation model evaluations can be very expensive and each parameter draw requires multiple model evaluations as a large number of agents need to be simulated via the natural history model in order to produce a single simulated target without too much stochastic variability. The exact time of each iteration is problem-specific, as it depends on the number of targets and how expensive they are to simulate. However, in this application, the start from scratch approach required 70.6 h, whereas the sequential approach required 48.1 h. Thus the sequential approach is significantly more efficient requiring only 68% of the time required by the start from scratch approach.

**Fig. 1:**
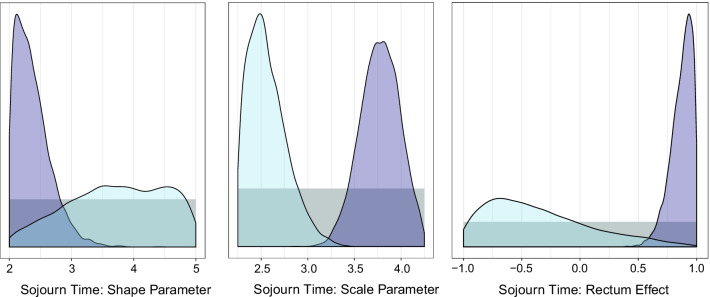
Posterior distributions of sojourn time parameters from the original calibration (light blue) and the recalibrated model that is calibrated to two additional targets using the start from scratch method (purple). Prior distributions are shown in gray

In Table 1, posterior means and 95% credible intervals are displayed for the model parameters for sojourn time from each of the three calibration results. (Results for all parameters are included in the “Appendix”.) Comparing the posterior estimates shown in Table 1 and the overlap or similarity measures based on $p_{orig}(\theta \mid y)$ and $p_{scratch}(\theta \mid y,z)$ in Table 2 indicates that the estimates of the parameters of the sojourn time function are significantly changed by the addition of new targets *z*. The scale parameter and rectal location effect for the distribution of sojourn time essentially do not overlap at all, and the posterior means are very different. In Fig. 1, we compare the marginal posterior distributions for the three sojourn time parameters from $p_{orig}(\theta \mid y)$ and $p_{scratch}(\theta \mid y,z)$. The posterior distribution for the shape parameter and rectal location effect for sojourn time become more peaked as a consequence of the more precise information provided by the new screen-detection rate targets. The sojourn time parameters are all meaningfully shifted in terms of location.

**Table 1:** Posterior means and 95% credible intervals of model parameters from original calibration to targets *y*, and from the model that is recalibrated to $\{y,z\}$ using a start from scratch approach as well as a sequential calibration approach

Parameter	Original posterior	New targets (start from scratch)	New targets (sequential)
Sojourn time			
$\lambda _2$	3.72 (2.20, 4.92)	2.38 (2.02, 3.07)	2.42 (2.02, 3.23)
$\lambda _1$	2.57 (2.27, 3.06)	3.77 (3.35, 4.16)	3.80 (3.36, 4.18)
$\lambda _3$	− 0.35 (− 0.96, 0.67)	0.87 (0.65, 0.99)	0.86 (0.61, 0.99)

Refer to  “The microsimulation model for colorectal cancer” section for details on model component distributions and parameters

**Table 2:** Credible interval overlap measures, area overlap measures, standardized differences in means (SMD), and Hellinger distances based on posterior samples from the original calibrated model ($p_{orig}(\theta \mid y)$), the start from scratch method for recalibration that incorporates new screen-detection rate targets ($p_{scratch}(\theta \mid y,z)$) and the sequential recalibration method that starts from the original calibrated posterior samples when adding in the new target ($p_{seq}(\theta \mid y,z)$)

	$p_{orig}(\theta \mid y)$ and $p_{scratch}(\theta \mid y,z)$				$p_{seq}(\theta \mid y,z)$ and $p_{scratch}(\theta \mid y,z)$			
Parameter	CI overlap	Area overlap	SMD	Hellinger	CI overlap	Area overlap	SMD	Hellinger
Sojourn time								
$\lambda _2$	0.57	0.14	1.75	0.70	0.93	0.88	0.06	0.07
$\lambda _1$	0.00	0.01	5.76	0.98	0.98	0.90	0.11	0.05
$\lambda _3$	0.04	0.04	2.76	0.90	0.96	0.92	0.02	0.05

**Fig. 2:**
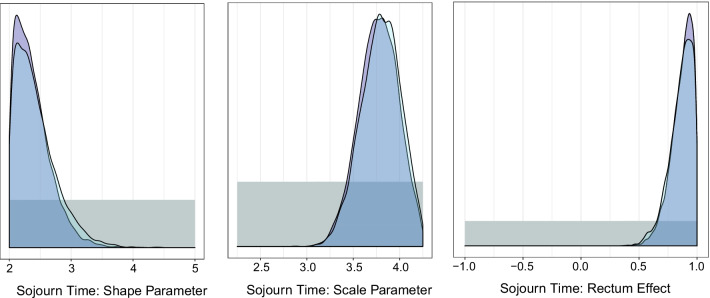
Posterior distributions for sojourn time parameters using the start from scratch method for recalibrating the model to fit new targets (purple) and the sequential calibration approach (light blue). Prior distributions are shown in gray

We also compare the two methodological approaches to recalibrating the model to match the new, more precise, study targets. The start from scratch approach and the sequential approach yield posterior distributions $p_{seq}(\theta \mid y,z)$ and $p_{scratch}(\theta \mid y,z)$ that are very similar. In Fig. 2, we compare the posterior distributions for the three sojourn time parameters previously shown, noting the similarity in the estimated distributions, which is expected given that both methods should be approximating the same posterior distribution, albeit using different approaches to get to that place. Given these findings, the sequential approach to recalibrating the model is preferred, as it takes far fewer iterations to reach convergence.

**Fig. 3:**
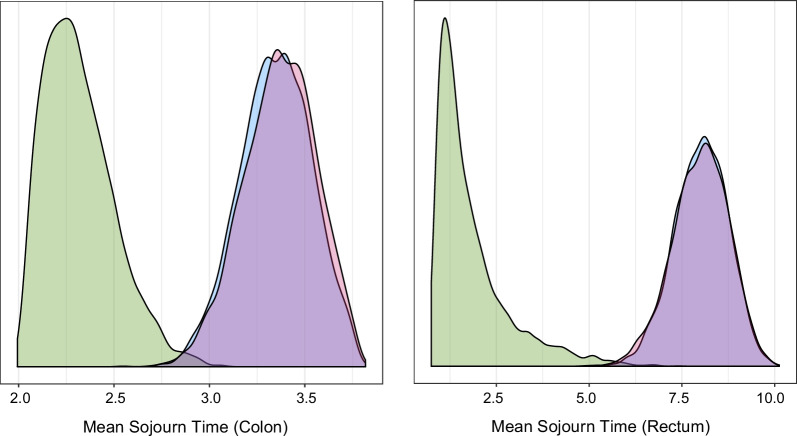
Posterior distributions for mean sojourn time under original calibration (green), and with new targets calibrated via the start from scratch method (blue) and the sequential calibration approach (pink). The overlap between the two methods for recalibration with new targets is shown in purple

While it is clear that the new screen-detection rate targets change the location and shape of the posterior distributions for the sojourn time distribution parameters, in order to more fully understand the implications of these changes on our estimates of sojourn time and cancer screening detection rates, we compute mean sojourn time (MST) for cancers in the colon and the rectum. MST is defined as $\lambda _1\Gamma (1+1/\lambda _2)$ for cancers in the colon and $\lambda _1\exp (\lambda _3)\Gamma (1+1/\lambda _2)$ for cancers in the rectum, where $\Gamma$ represents the gamma function. The posterior mean and 95% credible intervals for MST for cancers in the colon based on the original calibrated model that includes the imprecise screen-detection rate target of Imperiale et al. [11] are 2.32 (2.05, 2.74), whereas when calibrated to the more precise and gender-specific UKFSS targets (using the start from scratch method) they are 3.35 (2.97, 3.70). The posterior mean and 95% credible intervals for MST for cancers in the rectum based on the original calibration are 1.83 (0.85, 4.65), and calibrating to the UKFSS targets yields 8.01 (6.52, 9.33). Therefore, the more precisely estimated UKFSS targets significantly increase the estimated sojourn time for both types of cancers. This is a desirable outcome given that prior model validation work [40, 43] had suggested that CRC-SPIN underestimated the length of the preclinical cancer phase, providing sojourn time estimates that were too short per validation results and comparison to other competing models [16, 49]. Additionally, as expected, the sequential recalibration method yielded estimates for MST that were virtually identical to the start from scratch method: 3.37 (2.98, 3.71) for the colon and 8.00 (6.38, 9.37) for the rectum. Figure 3 compares posterior distributions for MST for cancers in the colon and rectum across all three sets of results. As expected, the posterior distributions from the recalibrated models that include the additional gender-specific screen-detection rate targets look virtually identical regardless of whether the start from scratch or sequential method is applied.

While each of the three calibrations provides a set of posterior samples of model parameters, targets are also simulated from the models during the process of model fitting. Since the models were calibrated to these targets, the simulated targets all “fit” the observed targets, however they may not be identical since the observed targets are estimated with error and therefore simulated targets are accepted as long as they fall within a specified tolerance interval of the observed targets (refer to the IMABC algorithm of Rutter et al. [43] for details). We therefore compare simulated targets (rather than model parameters) across the three sets of results. Tables 3 and 4 provide the overlap and standardized difference in means measures used to compare the original calibrated model to the updated model that incorporates the new screen-detected cancer rates, and to compare the two methods for recalibration. There are some simulated targets that change when the more precisely estimated screen detection rates are added as calibration targets. The most notable differences are in the number of preclinical cancers per large lesion [2, 21]. The number of preclinical CRCs per 1,000 adenomas greater than 10mm was estimated as 16 (95% CI of (13, 22)), but with the addition of new targets, these estimates increased to 33 (95% CI of (28, 37)) (identical under the start from scratch and sequential approach to calibration).

**Table 3:** Credible interval overlap measures, area overlap measures, and standardized differences in means based on simulated calibration targets from published studies

	$p_{orig}(\theta \mid y)$ and $p_{scratch}(\theta \mid y,z)$			$p_{seq}(\theta \mid y,z)$ and $p_{scratch}(\theta \mid y,z)$		
Calibration target	CI overlap	Area overlap	SMD	CI overlap	Area overlap	SMD
Pickhardt et al. [33]*						
% of detected adenomas						
$\le 5$mm	0.80	0.59	0.11	0.95	0.92	0.03
$6-9$mm	0.81	0.57	0.57	0.93	0.93	0.06
$\ge 10$mm	0.70	0.33	1.12	0.96	0.89	0.06
Corley et al. [3]						
Prevalence, Men 50–54	0.62	0.25	1.39	0.91	0.76	0.21
Prevalence, Men 55–59	0.63	0.28	1.29	0.92	0.75	0.21
Prevalence, Men 60–54	0.69	0.33	1.11	0.92	0.77	0.21
Prevalence, Men 65–69	0.75	0.41	0.89	0.92	0.79	0.19
Prevalence, Men 70–74	0.84	0.53	0.62	0.93	0.81	0.17
Prevalence, Men 75+	0.85	0.65	0.41	0.93	0.84	0.15
Prevalence, Women 50–54	0.65	0.25	1.33	0.96	0.92	0.04
Prevalence, Women 55–59	0.62	0.27	1.30	0.97	0.89	0.06
Prevalence, Women 60–54	0.64	0.32	1.18	0.98	0.90	0.07
Prevalence, Women 65–69	0.69	0.40	0.96	0.97	0.92	0.05
Prevalence, Women 70–74	0.79	0.54	0.69	0.98	0.93	0.04
Prevalence, Women 75+	0.86	0.68	0.45	1.00	0.95	0.02
Church [2]						
Pre-CRCs per 1000 lesions						
[6, 10)mm	0.65	0.30	1.39	0.98	0.92	0.08
$\ge 10$mm	0.00	0.00	6.70	0.97	0.91	0.09
Lieberman et al. [21]*						
Pre-CRCs per 1000 lesions						
$6-9$mm	0.90	0.73	0.36	0.96	0.93	0.07
$\ge 10$mm	0.00	0.00	7.68	0.98	0.92	0.03

We compare the original calibrated model ($p_{orig}(\theta \mid y)$), the start from scratch method for recalibration that incorporates new screen-detection rate targets ($p_{scratch}(\theta \mid y,z)$) and the sequential recalibration method that starts from the original calibrated posterior samples when adding in the new target ($p_{seq}(\theta \mid y,z)$). $^*$Size was reported categorically as $\le 5$mm, 6 to 9mm, and $\ge 10$mm. We operationalized these categories as: [1, 5.5) mm, [5.5, 9.5) mm and $\ge 9.5$ mm

**Table 4:** Credible interval overlap measures, area overlap measures, and standardized differences in means based on simulated annual incidence of clinically detected cancers in 1975–1979 (e.g., SEER calibration targets) [27]

	$p_{orig}(\theta \mid y)$ and $p_{scratch}(\theta \mid y,z)$			$p_{seq}(\theta \mid y,z)$ and $p_{scratch}(\theta \mid y,z)$		
Clinically detected cancer incidence, 1975–1979	CI overlap	Area overlap	SMD	CI overlap	Area overlap	SMD
Colon, female, 20–49	0.94	0.76	0.31	0.98	0.95	0.02
Colon, female, 50–59	0.95	0.82	0.25	0.98	0.94	0.05
Colon, female, 60–69	0.98	0.91	0.05	0.99	0.95	0.03
Colon, female, 70–84	0.92	0.66	0.48	1.00	0.95	0.02
Colon, female, 85+	0.92	0.70	0.44	0.99	0.92	0.09
Colon, male, 20–49	0.93	0.76	0.33	1.00	0.94	0.03
Colon, male, 50–59	0.96	0.88	0.16	0.98	0.91	0.08
Colon, male, 60–69	0.99	0.92	0.08	0.97	0.90	0.12
Colon, male, 70–84	0.90	0.66	0.47	0.97	0.89	0.12
Colon, male, 85+	0.87	0.64	0.55	0.97	0.94	0.04
Rectal, female, 20–49	0.89	0.65	0.55	1.00	0.94	0.03
Rectal, female, 50–59	0.97	0.88	0.11	0.98	0.91	0.10
Rectal, female, 60–69	0.95	0.78	0.30	0.98	0.89	0.14
Rectal, female, 70–84	0.93	0.65	0.52	0.98	0.89	0.14
Rectal, female, 85+	0.91	0.73	0.38	0.96	0.93	0.09
Rectal, male, 20–49	0.98	0.91	0.10	0.98	0.93	0.06
Rectal, male, 50–59	0.84	0.49	0.86	0.99	0.94	0.05
Rectal, male, 60–69	0.86	0.53	0.79	0.98	0.94	0.07
Rectal, male, 70–84	0.93	0.77	0.34	0.99	0.92	0.10
Rectal, male, 85+	0.97	0.92	0.03	0.98	0.92	0.07

We compare the original calibrated model ($p_{orig}(\theta \mid y)$), the start from scratch method for recalibration that incorporates new screen-detection rate target ($p_{scratch}(\theta \mid y,z)$) and the sequential recalibration method that starts from the original calibrated posterior samples when adding in the new target ($p_{seq}(\theta \mid y,z)$)

## Discussion

We have proposed a method for recalibrating a microsimulation model to incorporate new calibration targets. This method can be used when new information becomes available that requires the original targets to be supplemented with additional targets. We applied the algorithm to recalibrate a natural history model for CRC to include additional targets based on larger studies, and found that it was more efficient than starting over from scratch.

There are other settings in which this method is useful for recalibration, not just when adding new calibration targets. Sensitivity of colonoscopy is a fixed model input (non-calibrated parameter) that is specified based on direct evidence. A recent meta-analysis [52] examining the sensitivity of colonoscopy indicated that the sensitivity we assumed for a colonoscopy exam, which was based on earlier studies [10, 38], may have been too high. Test sensitivity is a critical input for any simulations of screening effects, since it determines whether an adenoma of a given size is detected and removed upon screening. Information from this recent meta-analysis suggests we consider re-specifying the probability of not detecting an adenoma to incorporate lower test sensitivities for small adenomas. We used the sequential approach to recalibration to generate posterior samples under a lower test sensitivity value for small adenomas. That is, we began with the posterior samples obtained from calibration to the original, higher, assumed sensitivity, since evidence from “Recalibrating the microsimulation model to incorporate new study targets” section indicates this can speed up the calibration process relative to starting over from scratch. While the original calibration based on initially specified test sensitivity inputs required 73 iterations, this process ultimately requires only another 24 algorithm iterations to achieve an ESS that exceeds 5000.

This work suggests that modelers may be able to substantially speed up the time needed to recalibrate their models. In many MSM or agent based modeling applications, calibration is a challenging and time consuming process due to the stochastic nature of the models and the need for embedded simulations to evaluate the computationally expensive model a large number of times (e.g., [25]). This prevents modelers from fully exploring the sensitivity of the model to inputs, or assumptions, and the choice of calibration data. Thus, a model is typically only calibrated once, and that calibrated model is ultimately used for making predictions about population trends in disease outcomes and effectiveness of interventions. The methods we propose here reduce the computing and person time required to recalibrate a model, and therefore aid in the ability of modelers to more fully explore sensitivity to assumptions and make changes to data or inputs when the evidence changes. Furthermore, in modeling applications where new data are continuously made available through, for example daily or weekly case counts for infectious diseases, the sequential approach becomes even more relevant for tracking the changing underlying contexts in a timely manner by efficiently generating updated parameter estimates.

While we focused on speeding up calibration within an approximate Bayesian framework, another promising approach to efficient calibration includes the use of an emulator (e.g., [7, 45]). An emulator is statistical representation of the MSM model that is less expensive to compute. The MSM can be evaluated at a series of design points, and the emulator can then be “trained” to fit these simulated outputs. Developing an emulator for computationally expensive MSMs is appealing, however the emulator would have to be complex and accurate enough to fit the model well across the entire parameter space, which is difficult in high-dimensional settings. This represents a promising area for future research.

While the methods developed here apply to situations when additional calibration data is included as a supplement to the original calibration data, future work will focus on settings where the original calibration targets are not a subset of the updated set. That is, when one wants to remove some of the original targets completely, possibly replacing them with other targets that are inconsistent with the original set. More generally, we aim to understand more fully when the sequential recalibration approach proposed here that builds on existing posterior samples does not converge to the correct posterior distribution, or is not more efficient than starting over from scratch.

## Conclusion

We proposed a method for recalibrating a MSM that can be used when new information becomes available that requires the original calibration targets to be supplemented with new targets. We applied the algorithm to recalibrate a natural history model for CRC to include additional targets based on larger studies because model examination and validation indicated that more information was needed to inform sojourn time. We found that the sequential approach to calibration was more efficient than starting over from scratch. The new targets resulted in changes in the posterior distribution for mean sojourn time, suggesting longer sojourn times than previously estimated.

### Supplementary Information

**Additional file 1:** Description of the Incremental Mixture Approximate Bayesian Computation Algorithm.

## Data Availability

The datasets used and/or analysed during the current study available from the corresponding author on reasonable request.

## Appendix

Table 5 displays posterior means and 95% credible intervals for all model parameters from each of the three calibration results. Table 6 displays the overlap or similarity measures based on $p_{orig}(\theta \mid y)$ and $p_{scratch}(\theta \mid y,z).$

**Table 5:** Posterior means and 95% credible intervals of model parameters from original calibration to targets *y*, and from the model that is recalibrated to $\{y,z\}$ using a start from scratch approach as well as a sequential calibration approach

Parameter	Original posterior	New targets (start from scratch)	New targets (sequential)
Adenoma risk			
*A*	− 6.56 (− 6.89, − 6.11)	− 6.49 (− 6.76, − 6.10)	− 6.47 (− 6.72, − 6.11)
$\alpha _1$	− 0.62 (− 0.70, − 0.49)	− 0.65 (− 0.70, − 0.56)	− 0.66 (− 0.70, − 0.60)
$\sigma _{\alpha }$	1.62 (1.43, 1.74)	1.69 (1.59, 1.75)	1.69 (1.57, 1.75)
$\alpha _{20}$	0.04 (0.03, 0.05)	0.04 (0.04, 0.05)	0.04 (0.04, 0.05)
$\alpha _{50}$	0.04 (0.02, 0.05)	0.03 (0.02, 0.05)	0.03 (0.01, 0.05)
$\alpha _{60}$	0.02 (− 0.01, 0.05)	0.01 (− 0.01, 0.04)	0.01 (− 0.01, 0.04)
$\alpha _{70}$	0.00 (− 0.02, 0.02)	0.00 (− 0.02, 0.03)	0.00 (− 0.02, 0.03)
Adenoma growth			
$\beta _{2c}$	37.67 (34.02, 39.86)	35.34 (31.96, 38.79)	35.42 (32.26, 38.75)
$\beta _{1c}$	1.43 (1.162, 1.701)	1.59 (1.31, 1.83)	1.58 (1.31, 1.81)
$\beta _{2r}$	15.28 (11.81, 19.53)	14.17 (11.29, 18.14)	14.14 (11.30, 17.82)
$\beta _{1r}$	3.39 (1.97, 4.76)	3.24 (1.80, 4.82)	3.39 (1.85, 4.83)
*p*	0.69 (0.56, 0.83)	0.79 (0.65, 0.92)	0.79 (0.66, 0.92)
Transition to preclinical CRC			
$\gamma _0$	3.16 (3.03, 3.35)	3.17 (3.08, 3.26)	3.17 (3.10, 3.26)
$\gamma _1$	− 0.13 (− 0.19, − 0.07)	− 0.13 (− 0.18, − 0.08)	− 0.13 (− 0.18, − 0.09)
$\gamma _2$	− 0.05 (− 0.24, 0.13)	− 0.04 (− 0.25, 0.14)	− 0.04 (− 0.23, 0.13)
$\gamma _3$	0.07 (− 0.03, 0.17)	0.11 (0.01, 0.20)	0.10 (0.01, 0.19)
$\gamma _4$	− 1.11 (− 1.42, − 0.79)	− 0.74 (− 1.08, − 0.37)	− 0.75 (− 1.07, − 0.41)
$\gamma _5$	0.12 (0.01, 0.23)	0.10 (0, 0.23)	0.10 (− 0.001, 0.23)
$\sigma _{\gamma }$	0.56 (0.50, 0.65)	0.52 (0.50, 0.55)	0.52 (0.50, 0.56)
Sojourn time			
$\lambda _2$	3.72 (2.20, 4.92)	2.38 (2.02, 3.07)	2.42 (2.02, 3.23)
$\lambda _1$	2.57 (2.27, 3.06)	3.77 (3.35, 4.16)	3.80 (3.36, 4.18)
$\lambda _3$	− 0.35 (− 0.96, 0.67)	0.87 (0.65, 0.99)	0.86 (0.61, 0.99)

Refer to “The microsimulation model for colorectal cancer” section for details on model component distributions and parameters

**Table 6:** Credible interval overlap measures, area overlap measures, standardized differences in means (SMD), and Hellinger distances based on posterior samples from the original calibrated model ($p_{orig}(\theta \mid y)$), the start from scratch method for recalibration that incorporates new screen-detection rate targets ($p_{scratch}(\theta \mid y,z)$) and the sequential recalibration method that starts from the original calibrated posterior samples when adding in the new target ($p_{seq}(\theta \mid y,z)$)

	$p_{orig}(\theta \mid y)$ and $p_{scratch}(\theta \mid y,z)$				$p_{seq}(\theta \mid y,z)$ and $p_{scratch}(\theta \mid y,z)$			
Parameter	CI overlap	Area overlap	SMD	Hellinger	CI overlap	Area overlap	SMD	Hellinger
Adenoma risk								
*A*	0.91	0.69	0.35	0.21	0.97	0.86	0.07	0.08
$\alpha _1$	0.83	0.58	0.57	0.24	0.86	0.73	0.24	0.16
$\sigma _{\alpha }$	0.72	0.41	0.87	0.40	0.95	0.93	0.04	0.05
$\alpha _{20}$	0.97	0.90	0.11	0.07	0.98	0.94	0.04	0.03
$\alpha _{50}$	0.90	0.68	0.51	0.17	0.98	0.94	0.04	0.03
$\alpha _{60}$	0.92	0.54	0.71	0.24	0.99	0.91	0.08	0.04
$\alpha _{70}$	0.93	0.70	0.44	0.16	1.00	0.92	0.09	0.03
Adenoma growth								
$\beta _{2c}$	0.76	0.32	1.47	0.45	0.97	0.92	0.05	0.04
$\beta _{1c}$	0.75	0.39	1.18	0.40	0.98	0.88	0.12	0.06
$\beta _{2r}$	0.87	0.61	0.57	0.21	0.98	0.95	0.01	0.04
$\beta _{1r}$	0.96	0.81	0.20	0.10	0.99	0.85	0.20	0.07
*p*	0.66	0.31	1.44	0.48	0.97	0.93	0.01	0.05
Transition to Pre CRC								
$\gamma _0$	0.78	0.55	0.06	0.29	0.96	0.90	0.05	0.06
$\gamma _1$	0.88	0.77	0.05	0.14	0.97	0.92	0.03	0.05
$\gamma _2$	0.99	0.87	0.11	0.06	0.97	0.92	0.03	0.05
$\gamma _3$	0.83	0.57	0.70	0.25	0.97	0.84	0.19	0.08
$\gamma _4$	0.44	0.18	2.24	0.66	0.96	0.90	0.04	0.05
$\gamma _5$	0.97	0.74	0.34	0.13	0.99	0.92	0.08	0.04
$\sigma _{\gamma }$	0.63	0.29	1.11	0.57	0.94	0.86	0.07	0.08
Sojourn time								
$\lambda _2$	0.57	0.14	1.75	0.70	0.93	0.88	0.06	0.07
$\lambda _1$	0.00	0.01	5.76	0.98	0.98	0.90	0.11	0.05
$\lambda _3$	0.04	0.04	2.76	0.90	0.96	0.92	0.02	0.05

## Abbreviations

ABC: Approximate Bayesian computation
CMS: Centers for Medicare and Medicaid Services
CRC: Colorectal cancer
CRC-SPIN: ColoRectal Cancer Simulated Population model for Incidence and Natural history
IMABC: Incremental Mixture Approximate Bayesian Computation
MCMC: Markov chain Monte Carlo
MSMs: Microsimulation models
MST: Mean sojourn time
SEER: Surveillance, epidemiology, and end results
SMD: Standardized difference in means
UKFSS: United Kingdom Flexible Sigmoidoscopy Screening
