# Supplementary material for: Sequentially calibrating a Bayesian microsimulation model to incorporate new information and assumptions
Source: BMC Med Inform Decis Mak. 2022 Jan 12;22:12. doi: 10.1186/s12911-021-01726-0 (PMC8756687; doi:10.1186/s12911-021-01726-0)
Supplement: Supplementary file 1 — Additional file 1: Description of the Incremental Mixture Approximate Bayesian Computation Algorithm. [file 12911_2021_1726_MOESM1_ESM.pdf]

Supplementary Material for "Sequentially Calibrating a Bayesian Microsimulation Model to Incorporate New Information and Assumptions"

Maria DeYoreo, Carolyn M. Rutter, Jonathan Ozik, Nicholson Collier

1 IMABC Algorithm

We refer the reader to Rutter et al. (2019) (Section 4) for a detailed description of the algorithm, but here provide a description of the algorithm and its most important features. The IMABC algorithm begins by generating model parameter vectors θ from the prior distribution, $\pi(\theta)$, like in the basic rejection-based ABC algorithm (Tavare et al., 1997; Pritchard et al., 1999). Sampled parameter vectors are accepted or rejected according to whether they result in simulated targets that are close to the observed calibration targets. An intersection criterion is used for acceptance (Conlan et al., 2012; Ratmann et al., 2014), such that a simulated parameter vector is only accepted if all simulated targets lie within the tolerance bounds, which define the level of discrepancy between simulated and observed targets that is deemed acceptable.

The tolerance bounds around the observed targets are governed by a parameter vector α, such that the tolerance bound around target j is based on a $(1-\alpha_j)\times 100\%$ confidence interval, which can be calculated since observed calibration targets are derived from published studies, and typically take the form of summary statistics with known distributions. The tolerance bounds are updated as the algorithm progresses. In the first iteration, very wide tolerance bounds can be used, with the more expensive to compute targets having $\alpha_j = 0$ so that fit to those targets does not need to be evaluated. The goal of these early iterations is simply to identify regions of the parameter space that produce acceptable fits to the easy to compute targets. This strategy is similar to the idea of History Matching (Craig et al., 1997; Andrianakis et al., 2017; Holden et al., 2018), which aims to identify parameter values that are implausible and thus to find viable regions of the parameter space for which the model outputs are consistent with the targets. Updating the tolerance bounds is important to allow the algorithm to find acceptable draws early on while ensuring that the final set of draws used for posterior inference is suitably close to the target distribution. Final α values used to calculate tolerance intervals are able to vary across targets, so that more precisely estimated targets can have narrower bounds than less precisely estimated targets. The algorithm then updates the initial sample by adding points near the set of "best" points. These are the points that result in simulated targets that are closest to corresponding observed targets. New draws are simulated at each iteration by sampling from a mixture of normal distributions, centered at the

current set of best points, with covariance matrices that reflect the correlation structure of the nearest, surrounding points. This strategy of drawing from multiple normal distributions at each iteration allows for parallel processing to be used while efficiently exploring the viable regions of the parameter space.

The process of accepting or rejecting sampled parameter vectors and updating the sample with new points is repeated, and at each step, it is checked to see if the tolerance intervals can be reduced in width by increasing the alpha-levels. The algorithm is repeated until the effective sample size (ESS) exceeds some minimum desired number (e.g., 5,000), and the alpha-levels reach the final user-specified alpha-levels. The ESS depends on how the information from the calibration will be used, where interval estimates of model outputs require fewer points than estimating functions of parameters, as we do here. Once the IMABC algorithm is complete, independent draws from the posterior distribution are simulated by taking a weighted sample from accepted points with replacement, using weights that account for simulation of draws from the posterior. ESS is the expected number of unique points obtained from the weighted samples. Refer to Rutter et al. (2019) (Section 4) for a detailed description of the algorithm and guidance on setting tuning parameters.

References

Andrianakis, I., McCreesh, N., Vernon, I., McKinley, T., Oakley, J. E., Nsubuga, R. N., and Goldstein, M. (2017), "History matching of a high dimensional HIV transmission individual based model," SIAM/ASA Journal of Uncertainty Quantification, 66, 694–719.

Conlan, A. J. K., McKinley, T. J., Karolemeas, K., Pollock, E. B., Goodchild, A. V., Mitchell, A. P., Birch, C. P. D., Clifton-Hadley, R. S., and Wood, J. L. N. (2012), "Estimating the Hidden Burden of Bovine Tuberculosis in Great Britain," PLOS Computational Biology, 8(10), 1–14.
URL: *https://doi.org/10.1371/journal.pcbi.1002730*

Craig, P. S., Goldstein, M., Seheult, A. H., and Smith, J. A. (1997), Pressure Matching for Hydrocarbon Reservoirs: A Case Study in the Use of Bayes Linear Strategies for Large Computer Experiments,, in Case Studies in Bayesian Statistics, eds. C. Gatsonis, J. S. Hodges, R. E. Kass, R. McCulloch, P. Rossi, and N. D. Singpurwalla, Springer New York, New York, NY, pp. 37–93.

Holden, P. B., Edwards, N. R., Hensman, J., and Wilkinson, R. D. (2018), "ABC for Climate: Dealing with Expensive Simulators," in Handbook of Approximate Bayesian Computation Chapman and Hall, pp. 1162–1173.
URL: *http://oro.open.ac.uk/56534/*

Pritchard, J. K., Seielstad, M. T., Perez-Lezaun, A., and Feldman, M. W. (1999), "Population growth of human Y chromosomes: a study of Y chromosome microsatellites.," Molecular Biology and Evolution, 16(12), 1791–1798.
URL: *+ http://dx.doi.org/10.1093/oxfordjournals.molbev.a026091*

Ratmann, O., Camacho, A., Meijer, A., and Donker, G. (2014), "Statistical modelling of summary values leads to accurate approximate Bayesian computations," ArXiv, arXiv:1305.4283.

Rutter, C., Ozik, J., DeYoreo, M., and Collier, N. (2019), "Microsimulation Model Calibration using Incremental Mixture Approximate Bayesian Computation," Annals of Applied Statistics, to appear.

Tavare, S., Balding, D., Griffiths, R., and Donnelly, P. (1997), "Inferring coalescence times from DNA sequence data," Genetics, 145, 505—518.
